# Distal Hypoperfusion Ischemic Syndrome in a Patient With End-Stage Renal Disease

**DOI:** 10.7759/cureus.74190

**Published:** 2024-11-21

**Authors:** Vivie Tran, Diego Olavarria-Bernal, Ana Cordón, Judy Lalmuanpuii

**Affiliations:** 1 Internal Medicine, Texas Tech University Health Sciences Center, Lubbock, USA

**Keywords:** chronic renal failure, dialysis, renal intervention, renal medicine, vascular surgery

## Abstract

Distal hypoperfusion ischemic syndrome (DHIS), also known as dialysis access steal syndrome (DASS), is a rare but significant complication in patients with end-stage renal disease (ESRD) undergoing hemodialysis through arteriovenous fistulas (AVFs). This case report presents a female patient in her 40s with a complex medical history, including peripheral arterial disease, coronary artery disease, and recurrent cellulitis affecting her right hand, who developed DHIS following the placement of a brachiobasilic AVF. Despite optimal medical management, the patient exhibited persistent ischemic symptoms, including hand coolness and necrosis, ultimately requiring surgical ligation of the AVF. Postoperatively, her symptoms significantly improved, highlighting the importance of early recognition and intervention in managing DHIS. This case demonstrates the need for a multidisciplinary approach involving nephrologists, vascular surgeons, and primary care providers to optimize patient outcomes and prevent severe complications. Furthermore, it emphasizes the necessity for standardized screening protocols for high-risk patients with AVFs, considering the psychosocial factors that can impact treatment adherence and long-term management.

## Introduction

Distal hypoperfusion ischemic syndrome (DHIS), also known as dialysis access steal syndrome (DASS), is a rare complication seen in patients with end-stage renal disease (ESRD) who undergo hemodialysis using arteriovenous fistulas (AVF) or grafts [1]. The pathophysiology of DHIS arises from arterial inflow that supplies two vascular beds with differing vascular resistances. This leads to the shunting of blood flow from the high-resistance bed (the distal extremity) to the low-resistance bed (the arteriovenous access). This can even result in a reversal of arterial flow, where blood flows from the distal extremity into the arteriovenous access [2]. Over time, such alterations in blood flow can lead to ischemia, manifesting in symptoms such as pain, coolness, and tissue necrosis [3].

Patients with ESRD often require hemodialysis as a life-sustaining therapy. AVF or grafts are commonly used for vascular access; however, they can lead to complications such as DHIS [4]. The incidence of DHIS is reported to be 1.9% in patients with AVF, with an increased risk in patients with small-caliber arteries or distal arterial disease [5].

Several additional risk factors have been identified, including advanced age, longer duration of dialysis treatment, and the presence of cardiovascular disease or diabetes mellitus. Patients diagnosed with DHIS are generally older and tend to have been on hemodialysis for an extended period compared to those without the syndrome [5].

Moreover, an elevated blood flow through the arteriovenous fistula and reduced oxygen saturation in the affected limb are linked to a higher risk of developing DHIS. Smoking has emerged as a significant predictor of survival in these patients, indicating its potential role in the onset or progression of the syndrome. Additionally, poor dialysis adequacy, along with inadequate erythropoietin therapy, is associated with worse outcomes in individuals with DHIS [5].

The management of DHIS is challenging and requires a multidisciplinary approach. The optimal treatment aims to alleviate ischemia while preserving the AVF. Although simple ligation is the most straightforward method to resolve this complication, it does result in the loss of the AVF. In cases where a patient develops ischemic muscle necrosis (IMN), immediate ligation of the AVF is essential. Treatment options for other DASS cases should be guided by factors such as the location of the anastomosis, the access flow volume, the condition of the feeding artery, and the clinical stage and severity of DHIS. Patients with mild DHIS typically resolve within a few weeks and benefit from close monitoring, hand warming, and physical therapy. However, surgical intervention with ligation of the AVF or graft is often necessary in severe or refractory cases [2].

Here, we present a case of a female in her 40s with ESRD who developed DHIS in the setting of a right arm brachiobasilic AVF. Despite optimal medical management, including antibiotics for recurrent cellulitis, the patient's symptoms persisted, ultimately requiring surgical ligation of the fistula. The aim of this case report is to depict an uncommon vascular complication and bring attention to conditions that remain in a gray zone between specialties such as internal medicine, nephrology, and vascular surgery, which should be immediately recognized.

## Case presentation

A female in her 40s with a history of peripheral arterial disease (PAD) status post right superficial femoral artery stent placement, coronary artery disease (CAD) status post coronary artery bypass grafting (CABG), hypertension (HTN), hyperlipidemia (HLD), ESRD secondary to diabetes mellitus (DM) on hemodialysis via a right brachiobasilic AVF, and bipolar disorder presented to the hospital with recurrent ulcers and cellulitis affecting her right hand. She had undergone multiple surgical procedures, including stent placements in both arms and legs and toe amputations due to infections with carbapenem-resistant *Pseudomonas* and vancomycin-resistant *Enterococcus* (VRE). The patient reported a small cut on her right 4th digit four months ago, initially treated with cephalexin without improvement. Subsequently, she was switched to trimethoprim/sulfamethoxazole, which provided temporary relief. However, the cellulitis recurred, leading to a course of clindamycin that proved ineffective, prompting a return to Bactrim for a 10-day course. Additionally, she noted worsening necrosis of the 2nd and 4th digits of her right hand for several months. Despite being on multiple antibiotic regimens, the patient's cellulitis persisted, indicating a need for further evaluation to determine the underlying etiology of her symptoms.

The patient reported hand coolness and cramping, which worsened during dialysis but improved with wearing a glove and dangling the hand. On examination, her hand presented erythema, swelling, and necrosis of the affected digits. She also presented decreased radial pulse in her right hand and dry skin. There was a 5 mm eschar on the right 4th proximal interphalangeal (PIP) joint and a 1 mm eschar on the right 2nd distal phalanx. Mild erythema was noted on the right digits 2-5, with no associated warmth. Tenderness to palpation was present, especially on the 4th and 2nd digits of the right hand. She presented multiple toe amputations in both feet. The cardiovascular exam revealed a regular rate and rhythm with reduced peripheral perfusion of the right hand. The respiratory exam was unremarkable.

**Figure 1:**
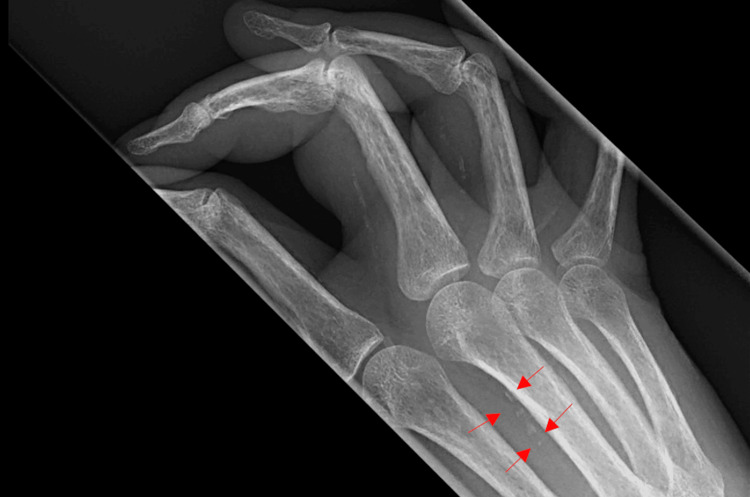
Calcification of peripheral vessels is evident on the hand X-ray lateral view (shown by arrows).

**Figure 2:**
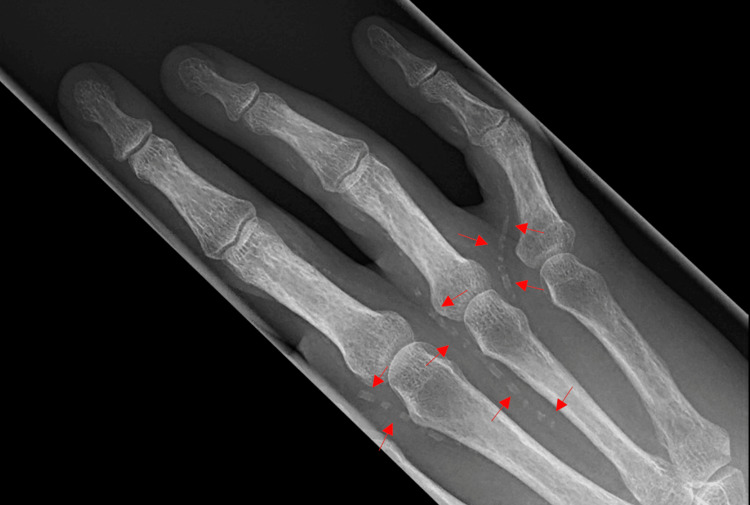
Calcification of peripheral vessels is evident on the hand X-ray superior view (shown by arrows).

During the initial workup, a hand MRI ruled out osteomyelitis. The hand X-ray showed calcified vessels (Figures 1, 2), and the Doppler studies confirmed steal syndrome affecting the extremity. Surgical ligation of the fistula was recommended as the definitive treatment, considering the patient's history of recurrent infections, worsening ischemia, and poor quality of life. After discussing the plan with the patient, she was transitioned to hemodialysis via a tunneled catheter, and the patient underwent ligation of the AVF two weeks later. The patient's symptoms improved following the procedure, with the resolution of ulcers and cellulitis. She was referred to the transplant team for consideration of renal transplantation to avoid further complications related to vascular access; however, she had been rejected twice in the past due to her poor functional status and uncontrolled bipolar disorder at the time of evaluation. Although her wounds had healed, she remained ineligible for kidney transplantation due to multi-organ dysfunction, as determined by the committee's decision.

## Discussion

DHIS, or DASS, is a rare but recognized complication of AVF or grafts used for hemodialysis in patients with ESRD. It occurs when blood flow is diverted from the distal vasculature to the access site, leading to ischemia of the distal extremity [1]. Patients with ESRD who develop DHIS often have additional risk factors that predispose them to this condition, including DM, PAD, HTN, and CAD [5]. Patients typically present with symptoms such as hand coolness, pain, and ischemic changes, as observed in our patient [3]. Diagnosis of DHIS is confirmed through noninvasive studies like digital photoplethysmography tracing, pressure measurements, and duplex ultrasound, which demonstrate the steal phenomenon [1].

If not treated timely and effectively, DHIS can lead to severe complications due to prolonged ischemia in the affected limb. Progressive ischemia results in tissue damage and necrosis, potentially necessitating amputation. Chronic ischemia can cause non-healing ulcers and gangrene, which are prone to infections that may escalate to systemic infections such as sepsis [6]. Functional impairment, including loss of limb function and dexterity, is common, and severe cases may require amputation to manage pain and prevent infection spread. Patients often experience chronic and neuropathic pain, which can significantly impact their quality of life. Vascular complications, including thrombosis and embolization, further compromise blood flow and pose life-threatening risks. Additionally, the psychosocial impact of chronic pain, functional limitations, and the threat of limb loss can lead to significant psychological distress, including depression and anxiety, further diminishing the patient's overall quality of life [7]. Thus, early recognition and prompt treatment of DHIS are crucial to prevent these severe and debilitating complications.

Management of DHIS includes conservative measures such as wearing gloves, maintaining hand warmth, and optimizing dialysis parameters to reduce access flow. In cases where conservative management fails, surgical intervention with ligation of the AV fistula or graft is necessary [8]. The outcomes for patients with DHIS can vary; notably, patients on hemodialysis who experienced DHIS had significantly reduced survival compared to those who did not have DHIS (160 months vs. 191 months) [5].

Several surgical interventions are available to treat DHIS, each with varying success rates. A study by Vajdič Trampuž et al. demonstrated that distal revascularization and interval ligation (DRIL) has an overall success rate of 81%, with a mean follow-up of 22.2 months. Proximal radial artery ligation (PRAL) shows a higher success rate of 89% but with a shorter mean follow-up of 13.8 months. Revision using distal inflow (RUDI) achieved an overall success rate of 82%, with a mean follow-up of 15.9 months. Proximalization of arterial inflow (PAI) boasts the highest success rate at 90%, though its mean follow-up period is shorter at 11.4 months. Banding also exhibits a commendable overall success rate of 87% with a mean follow-up of 11.6 months. Overall, the study concluded that all surgical interventions analyzed demonstrated high success rates in treating DHIS [9].

In our case, the patient presented with a right arm brachiobasilic AVF and exhibited symptoms of DHIS, including hand coolness, pain, and necrosis of the fingers. There was a delay of several months between the onset of initial symptoms and the final diagnosis. This delay underscores the importance of including DHIS in the differential diagnosis among nephrologists and primary care physicians to avoid irreversible complications and loss of function in the extremities.

Unfortunately, our patient was rejected for kidney transplantation due to bipolar disorder and poor adherence to post-transplant care, which raises concerns about her long-term management options. While a transplant may ultimately be the best solution for her condition, the chances of this occurring are minimal.

Moreover, it is crucial to note that while we cannot predict which patients will develop DHIS, we can identify risk factors. This emphasizes the need for proactive measures to recognize early symptoms and secure timely surgical evaluation. In summary, our patient’s symptoms improved significantly following surgical intervention, highlighting the potential effectiveness of this approach in resolving severe ischemic complications in selected cases. The critical need for a multidisciplinary approach to manage DHIS in patients with AVFs presenting with distal extremity ischemia cannot be overstated.

## Conclusions

Our case of a female in her 40s highlights the critical need for early recognition and prompt management of DHIS. Despite optimal medical treatment, surgical evaluation is often necessary and frequently represents the most effective approach to alleviate severe ischemic complications. This case highlights the importance of a multidisciplinary approach involving internists, nephrologists, and vascular surgeons to optimize patient outcomes and prevent permanent complications. Moreover, it emphasizes the urgent need for standardized screening protocols to identify high-risk patients with AVFs, particularly those with complex medical histories. Implementing routine assessments, including non-invasive vascular studies, could facilitate earlier diagnosis and intervention, ultimately improving patient prognosis and quality of life.

Additionally, our patient's rejection for kidney transplantation due to psychosocial factors highlights the importance of addressing mental health and adherence issues in chronic disease management. Future studies should explore the integration of psychological support and patient education into the care of individuals with ESRD to enhance adherence and outcomes. By fostering collaboration among specialties and prioritizing comprehensive care models, we can enhance management strategies for patients at risk of DHIS and similar complications, ultimately aiming for improved long-term outcomes and patient satisfaction.
